# Long-Read RNA Sequencing Reveals an Isoform Switching Pattern in Healthy Microglial Cell Lines Exposed to Radiotherapy

**DOI:** 10.3390/ncrna12040031

**Published:** 2026-08-06

**Authors:** Tatiana Zuppelli, Laura Orlando, Giuseppe N. Conti, Sofia P. Lombardo, Lucia Longhitano, Giulia Chisari, Cesarina Giallongo, Francesco Bellia, Maria Gabriella Sabini, Stefania Mele, Ignazio A. Barbagallo, Nunzio Vicario, Rosalba Parenti, Michelino Di Rosa, Enrico La Spina, Stefano Forte, Daniele Tibullo, Giovanni Li Volti

**Affiliations:** 1 Department of Biomedical and Biotechnological Sciences, University of Catania, 95123 Catania, Italy; tatiana.zuppelli@gmail.com (T.Z.); lauraorlando2810@gmail.com (L.O.); sofia.lombardo@phd.unict.it (S.P.L.); lucia.longhitano@unict.it (L.L.); cesarina.giallongo@unict.it (C.G.); francesco.bellia@unict.it (F.B.); ignazio.barbagallo@unict.it (I.A.B.); nunzio.vicario@unict.it (N.V.); parenti@unict.it (R.P.); mdirosa@unict.it (M.D.R.); d.tibullo@unict.it (D.T.); livolti@unict.it (G.L.V.); 2 Istituto Oncologico del Mediterraneo (IOM), 95029 Viagrande, Italy; giulia.chisari94@libero.it (G.C.); stefano.forte@grupposamed.com (S.F.); 3 Medical Physics Unit, Cannizzaro Hospital, 95126 Catania, Italy; mgabsabini@gmail.com (M.G.S.); mele.stefania@gmail.com (S.M.)

**Keywords:** long-read RNA sequencing, transcriptomic variants, radiotherapy, tumor microenvironment, computational biology, transcriptomic regulation

## Abstract

**Background/Objectives**: Radiotherapy (RT) is a fundamental treatment for brain tumors but often leads to long-term neurotoxicity, partly caused by microglial dysfunction. In this study, we used long-read RNA sequencing (LR-RNA-seq) to map the isoform-specific transcriptome of human microglial HMC3 cells after RT exposure. **Methods**: The HMC3 human microglial cell line was treated with standard radiotherapy and, after RNA extraction and library preparation, LR-RNA-seq was performed. Further bioinformatic analyses allowed to investigate on the isoform switching pattern after radiotherapy exposure. **Results**: Gene-level analysis found 591 differentially expressed genes, with positive enrichment of PI3K/AKT-related pathways and negative enrichment of translation-associated processes. Isoform-level analysis uncovered 827 significant isoform switching events across 420 genes, often involving shifts from protein-coding transcripts to non-coding variants. Functional annotation showed that most isoform switches resulted in the loss of open reading frames and protein domains, especially in ribosomal proteins and translation machinery components. Exploratory proteomic analysis identified 191 differentially abundant proteins, including a significant reduction in RPL7A, providing partial protein-level support for translation-associated transcriptomic remodeling. **Conclusions**: RT-induced stress causes qualitative transcriptomic remodeling beyond changes in overall gene expression. These findings indicate that radiotherapy induces coordinated gene-, transcript-, and isoform-level changes in HMC3 cells. The alterations observed in translation-associated genes, together with partial proteomic concordance, identify candidate mechanisms for future functional investigation.

## 1. Introduction

Traditional short-read RNA sequencing, while powerful, often struggles to fully characterize isoform diversity and detect complex structural variations in the transcriptome [1]. In contrast, long-read RNA sequencing (LR-RNA-seq) technologies offer a significant advantage by capturing full-length transcripts, thereby enabling comprehensive identification of novel isoforms, alternative splicing events, and gene fusions that are often missed by shorter reads [2,3]. This enhanced capability is particularly crucial in discerning the nuanced cellular responses to external stressors like ionizing radiation, which can profoundly impact transcriptional landscapes [4,5]. The ability to sequence entire mRNA molecules in a single read overcomes limitations of reference-based annotations and facilitates the discovery of rare or cell-type-specific isoforms that might be critical in understanding microglia’s response to radiation [2]. Furthermore, the high-throughput nature of next-generation sequencing, including long-read platforms, has catalyzed a new era of genomic research by enabling extensive and cost-effective analysis of DNA and RNA, thereby providing insights into gene expression, alternative splicing, and novel transcript identification [6].

This advancement is particularly relevant for microglial cells. Microglia serve as the resident innate immune cells of the central nervous system, playing a pivotal role in maintaining neural homeostasis, synaptic pruning, and the initial response to injury [2,7]. However, these cells are highly sensitive to environmental stressors, particularly ionizing radiation, which is a cornerstone of treatment for primary and metastatic brain tumors [8,9]. While radiotherapy (RT) is essential for controlling tumor growth, it often results in unintended radiation-induced brain injury, characterized by chronic neuroinflammation, oxidative stress, and cognitive decline, often referred to as “brain fog” [10,11]. In the context of the tumor microenvironment, irradiated microglia can undergo a phenotypic shift from a homeostatic state to an activated, often tumor-favorable state, which can promote radioresistance and glioma progression in the surrounding normal parenchyma [7,12,13].

Traditional transcriptomic studies of irradiated microglia have largely relied on short-read RNA sequencing. While these methods have identified “aging-like” signatures and inflammatory gene expression patterns in irradiated cells, they struggle to resolve the full complexity of the microglial transcriptome [14]. Short-read technologies often fail to fully characterize isoform diversity, as they cannot sequence full-length transcripts in a single pass, leading to ambiguities in detecting alternative splicing and structural variations [1,3]. This limitation is particularly significant because cellular stress response—such as that induced by radiotherapy—is often associated not only with changes in total gene expression, but with qualitative shifts in transcript usage, known as isoform switching [15,16].

Recent advancements in LR-RNA-seq, such as those provided by Oxford Nanopore and Pacific Biosciences, have revolutionized our ability to map the “isoform-centric” landscape of the brain [2,6]. By capturing entire mRNA molecules, LR-RNA-seq enables the comprehensive identification of novel isoforms and complex splicing events that are frequently missed by shorter reads [17,18]. These technologies have revealed that many genes respond to stress by switching from functional, coding isoforms to non-coding variants or those sensitive to nonsense-mediated decay, a process often termed “ribostress” or translational reprogramming [19,20]. Such isoform-level changes can result in a loss of functional protein domains, even when overall gene expression levels appear stable or upregulated [21,22].

Given the critical role of microglia in mediating both the therapeutic and adverse effects of radiotherapy, a high-resolution understanding of their transcriptomic response is essential. This study aims to characterize the complex modification of the transcriptomic landscape in human microglial cell lines following radiotherapy exposure. By utilizing LR-RNA-seq, we seek to identify specific isoform switching patterns and functional alterations that may drive microglial dysfunction and contribute to the remodeling of the brain tumor microenvironment after conventional radiotherapy. This detailed profiling is of critical importance for identifying novel biomarkers and therapeutic targets that could mitigate off-target effects of radiotherapy while potentially enhancing treatment efficacy.

## 2. Results

### 2.1. Radiotherapy-Treated Microglia Show Differential Expression Patterns at Both Gene and Transcript Levels

To profile gene-level transcriptional changes, transcript counts assigned to the same gene were aggregated and analyzed using DESeq2. Transcript-level differential abundance was analyzed using the same statistical framework, with each transcript treated as an independent molecular feature. Differentially expressed genes and differentially abundant transcripts were defined using an adjusted *p*-value < 0.05 and an absolute log2 fold change > 1.

**Figure 1:**
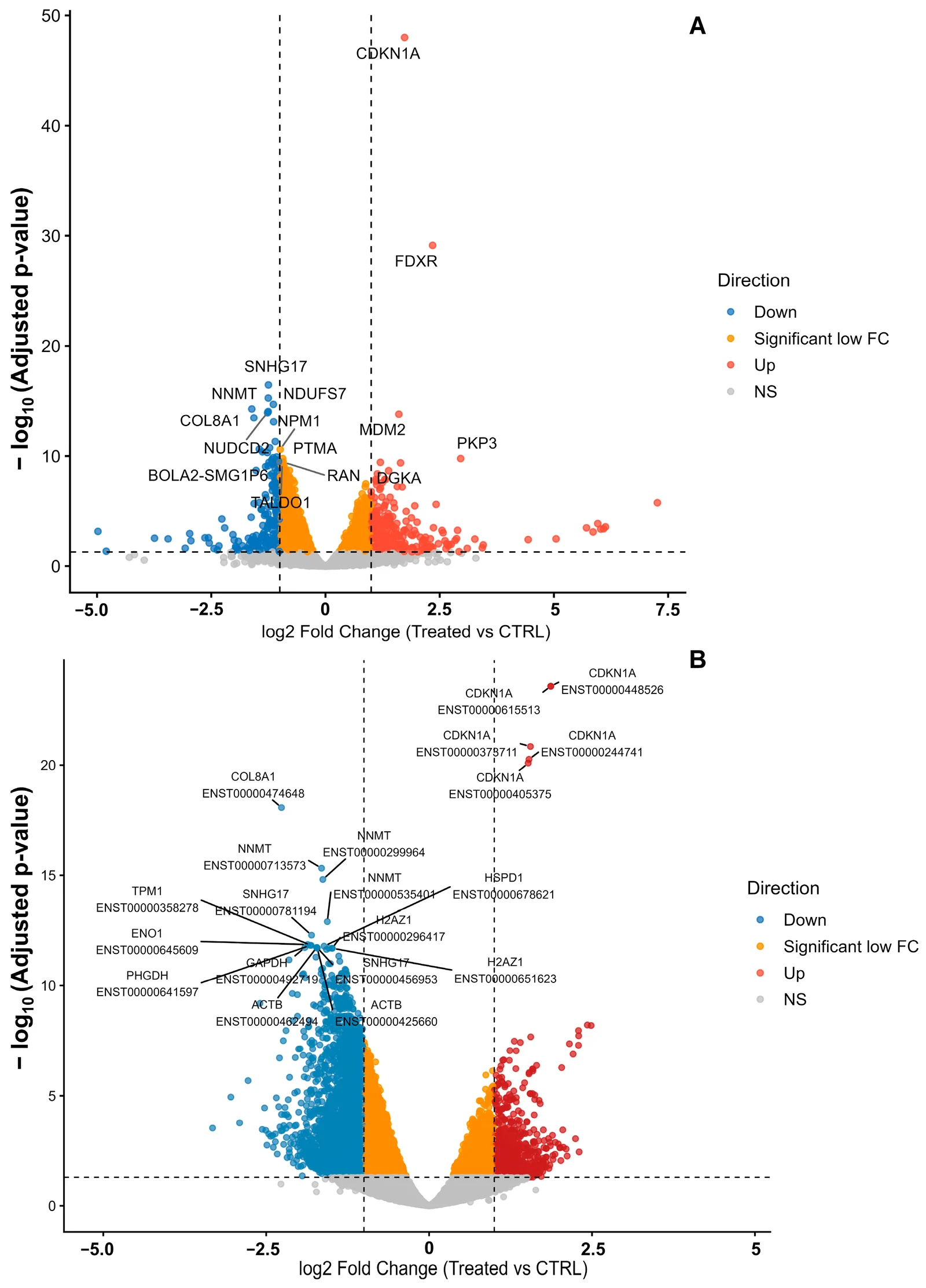
Volcano plots representing differentially expressed genes (**A**) and transcripts (**B**) between treated and control microglial cell lines. The *X*-axis reports the log2 Fold change in each gene between the two conditions. Statistical significance, indicated as −log10(FDR), is reported on the *Y*-axis. The dashed lines represent the statistical and biological cutoffs set, respectively: Adjusted *p*-value < 0.05; Absolute Log_2_ Fold Change > 1.

Among 11,116 genes, 591 were differentially expressed, including 220 downregulated and 371 upregulated genes (Figure 1A). At the transcript level, 3401 out of 53,662 transcripts were differentially abundant, including 839 upregulated and 2562 downregulated transcripts (Figure 1B).

Gene-level and transcript-level DESeq2 analyses are listed in Supplementary Tables S1 and S2, respectively.

### 2.2. Gene Set Enrichment Analysis Identifies Positive Enrichment of Signaling Pathways and Negative Enrichment of Translation-Related Processes

**Figure 2:**
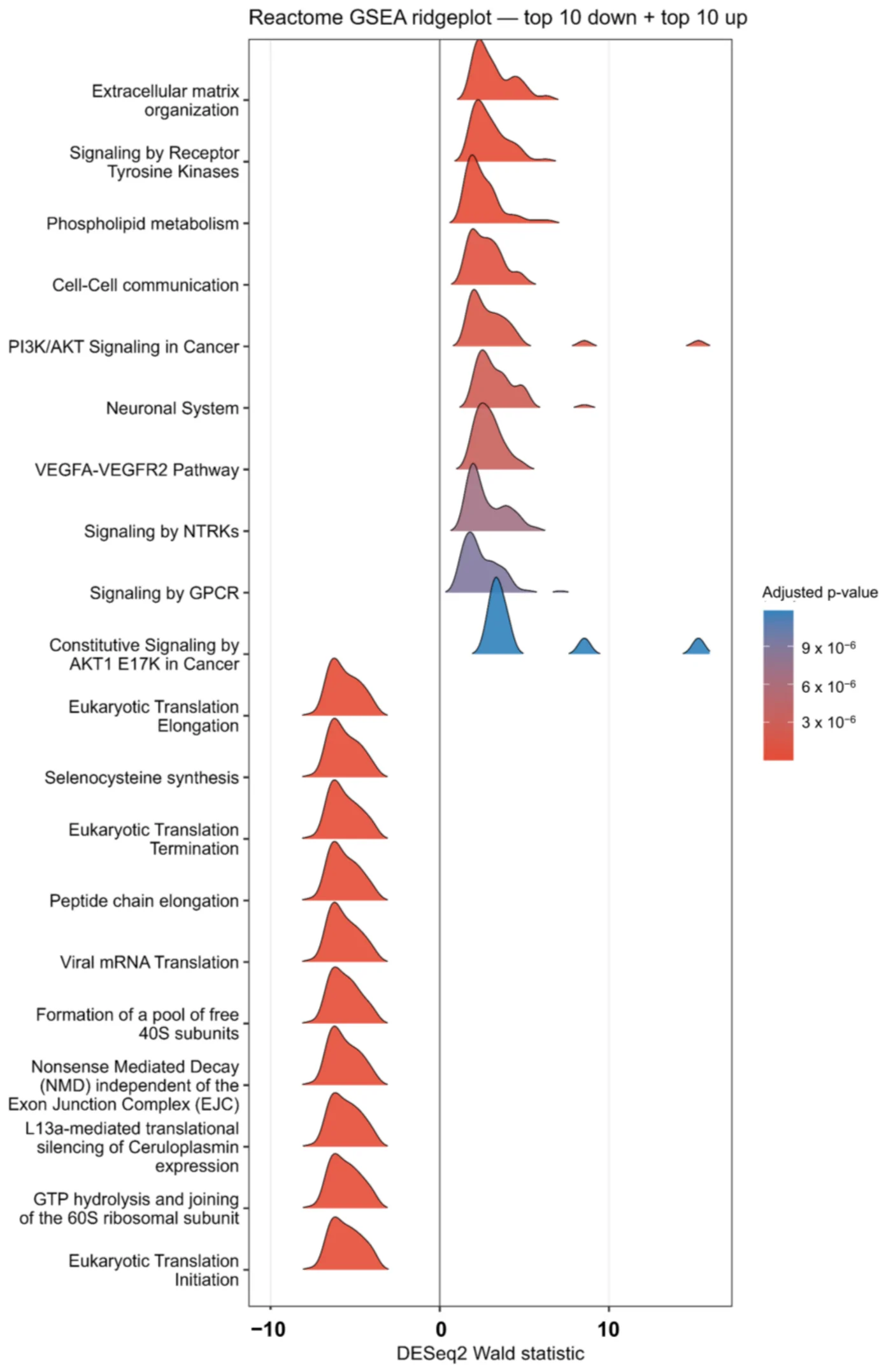
GSEA ridgeplot showing the distribution of the gene-level ranking metric across the most significantly enriched Reactome pathways in irradiated versus control microglial cells. The x-axis represents the DESeq2 Wald statistic used to rank genes for GSEA. Pathways enriched toward positive values are associated with genes relatively upregulated after radiotherapy, whereas pathways enriched toward negative values are associated with genes relatively downregulated after radiotherapy.

The Gene Set Enrichment Analysis (GSEA) performed on the complete gene-level ranked list allowed us to obtain the most affected pathways. The ten most positively enriched and ten most negatively enriched significant Reactome pathways are shown in the ridgeplot in Figure 2. Extracellular matrix organization and PI3K/AKT-related pathways showed positive normalized enrichment scores, indicating enrichment toward genes with higher expression in irradiated cells, whereas translation-related pathways showed negative enrichment scores.

### 2.3. Radiotherapy Induces Widespread Changes in Relative Isoform Usage

Using the IsoformSwitchAnalyzeR package (ver. 2.12.0), we performed an isoform-level analysis to detect potential switches from a canonical isoform to alternative variants. We identified 1364 isoform switches, of which 827 were significant (q < 0.05, |dIF| ≥ 0.1), spanning 420 genes. Specifically, considering normalized expression at both gene and isoform levels, we computed the Isoform Fraction (IF) as described in Section 4. This metric is crucial for determining isoform usage, that is, how much a given isoform contributes to its gene’s overall expression.

After calculating IF values for each condition, dIF was defined as IF_Treated − IF_CTRL. Positive dIF values indicate increased relative isoform usage in irradiated cells, whereas negative dIF values indicate reduced relative usage after RT.

To visualize the relationship between transcript-level differential abundance and isoform switching, we classified all isoforms according to their significance in the two analyses. This showed that 401 isoforms were supported by both significant isoform switching and transcript-level DESeq2 differential abundance, whereas 426 isoforms were significant only at the isoform-switching level (q < 0.05, |dIF| > 0.1). Conversely, 121 isoforms were significant only according to transcript-level DESeq2 (adjusted *p*-value < 0.05, |log2FC| > 1). These results are shown in Supplementary Figure S1.

**Figure 3:**
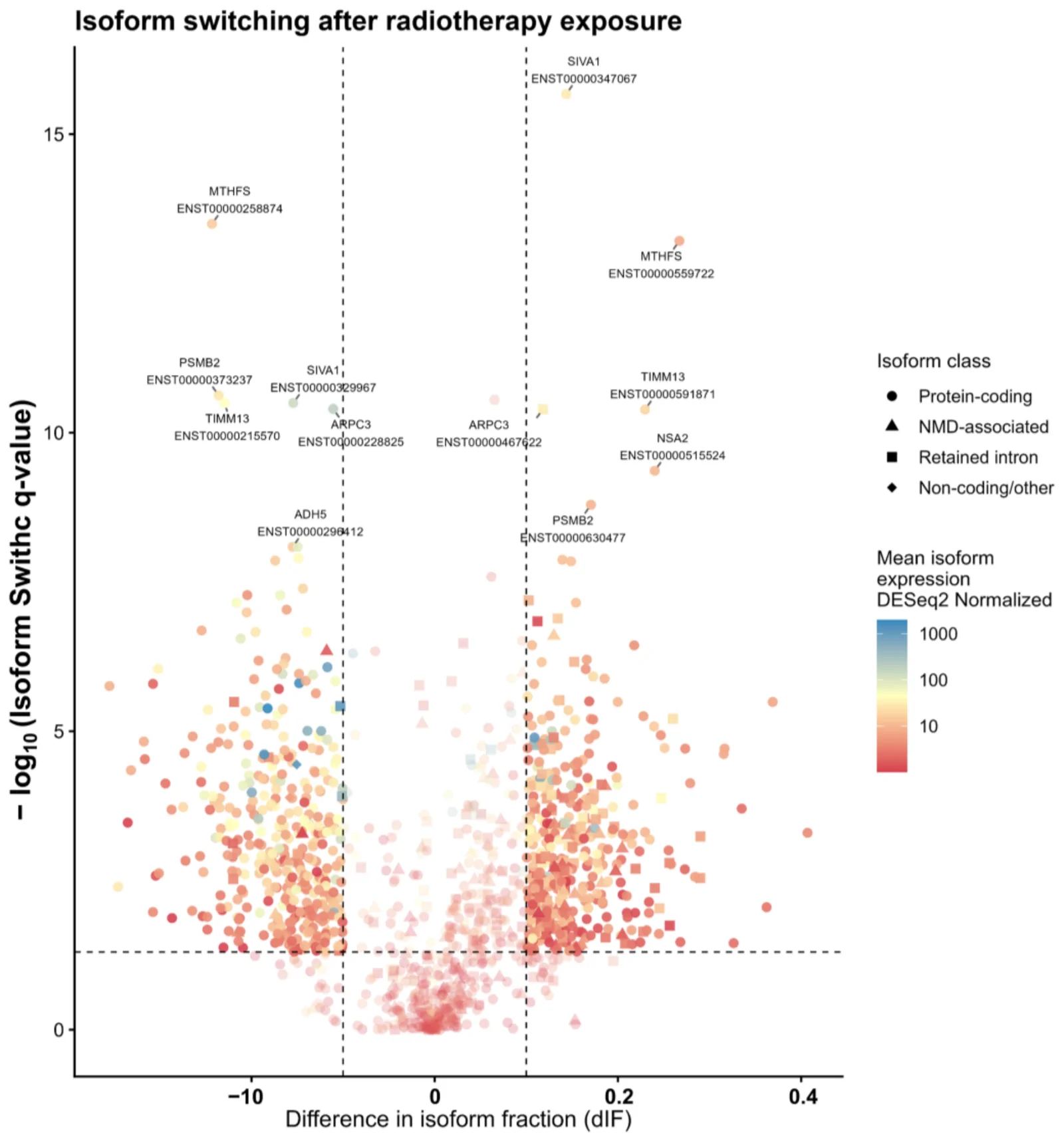
Isoform class and abundance of radiotherapy-associated isoform switches. Volcano plot showing isoform switching events according to the difference in isoform fraction (dIF) and isoform switch q-value. Dashed vertical lines indicate |dIF| = 0.10, while the dashed horizontal line indicates isoform switch q-value = 0.05. Point shape indicates transcript biotype-derived isoform class, whereas point color represents mean isoform expression across control and irradiated cells. Most significant isoforms are labeled with both gene symbol and transcript identifier.

Accordingly, all switches were displayed in a volcano plot (Figure 3) using statistical and biological cutoffs of q-value < 0.05 and |dIF| > 0.1, respectively.

The full list of significant switching genes and transcripts is provided in Supplementary Table S3.

### 2.4. Pathway Enrichment Analysis Identifies Cell-Cycle and Translation-Related Pathways Among Genes Affected by Isoform Switching

To investigate the biological processes associated with radiotherapy-induced isoform switching, we performed Reactome pathway enrichment analysis on genes carrying significant isoform-switching events. Because pathway annotations are assigned at the gene/protein level rather than at the individual isoform level, enrichment analysis was used to identify biological pathways over-represented among genes affected by isoform switching. The corresponding switched isoforms were then mapped back to the enriched pathways and stratified according to their direction of usage change and transcript biotype-derived isoform class.

**Figure 4:**
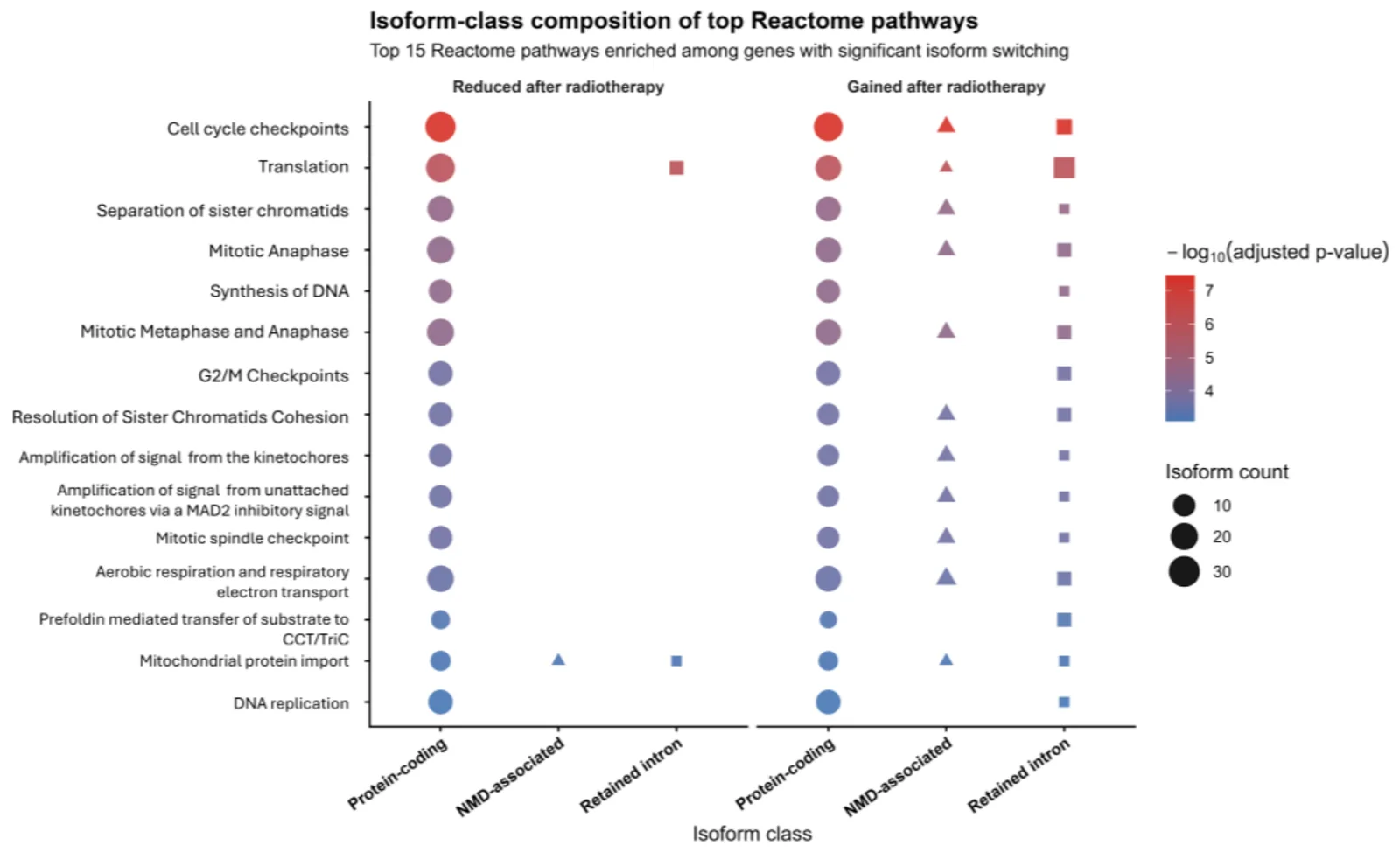
Isoform-class composition of the top Reactome pathways associated with significant isoform switching. The top 15 Reactome pathways were selected according to adjusted *p*-value among genes carrying significant isoform-switching events. For each pathway, switched isoforms belonging to pathway-associated genes were stratified according to whether their usage increased or decreased after radiotherapy. Point size indicates the number of switched isoforms in each isoform class, while point color indicates Reactome enrichment significance. Isoform classes were derived from transcript biotype annotation. The complete list of pathway-associated genes and switched isoforms is provided in Supplementary Table S4.

The top Reactome pathways enriched among genes with significant isoform switching were mainly related to cell-cycle regulation, mitotic progression, chromatid segregation, DNA replication, translation, mitochondrial respiration and mitochondrial protein import (Figure 4).

To avoid combining reciprocal isoform-switching events into a single undirected category, switched isoforms belonging to genes in the top enriched pathways were further separated according to whether their usage was reduced or gained after radiotherapy. This analysis showed that both reduced and gained isoforms contributed to the same major Reactome pathways, consistent with the reciprocal nature of isoform switching within genes involved in shared biological processes. Protein-coding isoforms represented the dominant class in both directions. However, gained isoforms after radiotherapy also included NMD-associated and retained-intron isoforms across several enriched pathways, particularly those related to cell-cycle checkpoints, mitotic progression, translation and mitochondrial processes.

### 2.5. Reactome GSEA Pathways Contain Genes Undergoing Coding and Non-Coding Isoform Remodeling

To integrate gene-level pathway enrichment with isoform-level regulation, we intersected Reactome GSEA core-enrichment genes with genes undergoing significant isoform switching. This analysis was designed to identify pathway-associated genes for which gene-level enrichment coexisted with changes in transcript isoform usage. Since Reactome GSEA is based on gene-level ranking and Reactome annotations are assigned at the gene/protein level, this overlap was interpreted as a prioritization strategy rather than as direct evidence of altered protein output.

Table 1 reports representative non-redundant Reactome pathways with positive or negative normalized enrichment scores (NES) and overlapping significant switching genes. Negative NES values indicate enrichment toward the downregulated side of the gene-level ranked list in treated versus control cells, whereas positive NES values indicate the opposite enrichment. For each pathway, Table 1 summarizes the transcript biotype-derived classes of isoforms showing increased or decreased relative usage after radiotherapy. The most recurrent overlaps were observed among negatively enriched pathways, including Translation, rRNA processing, Metabolism of amino acids and derivatives, DNA Replication, and mitochondrial respiratory pathways. These pathways contained multiple switching genes, including ribosomal proteins, translation initiation factors, mitochondrial ribosomal proteins, proteasome subunits, and respiratory chain-related genes. Although protein-coding isoforms represented the dominant class in both directions, several negatively enriched pathways also showed gained retained-intron isoforms, while respiratory electron transport-related pathways included gained NMD-associated isoforms.

Positively enriched pathways showed a more limited overlap with switching genes and were mainly represented by specific genes such as PLOD1 in extracellular matrix/collagen-related pathways, SHARPIN in neuronal or synapse-related pathways, and ID3 in NTRK signaling. In these cases, overlapping switched isoforms were classified as protein-coding in the compact pathway-level summary.

Representative genes were prioritized among significant isoform-switching genes that also belonged to the core-enrichment subset of significant Reactome GSEA pathways. Selection was based on pathway relevance, isoform-switch significance, magnitude of the isoform-fraction change, and the presence of transcript isoforms belonging to distinct biotype classes. RPS6, RPL4, RPL5, and RPL7A were selected as representative genes involved in negatively enriched translation- and ribosome-related pathways. PLOD1, SHARPIN, and ID3 were selected as representative switching genes associated with positively enriched non-translation pathways, including extracellular matrix organization, neuronal signaling, and NTRK signaling. These examples were used to illustrate distinct patterns of radiotherapy-associated isoform remodeling and were not intended to represent the complete set of significant switching events.

**Figure 5:**
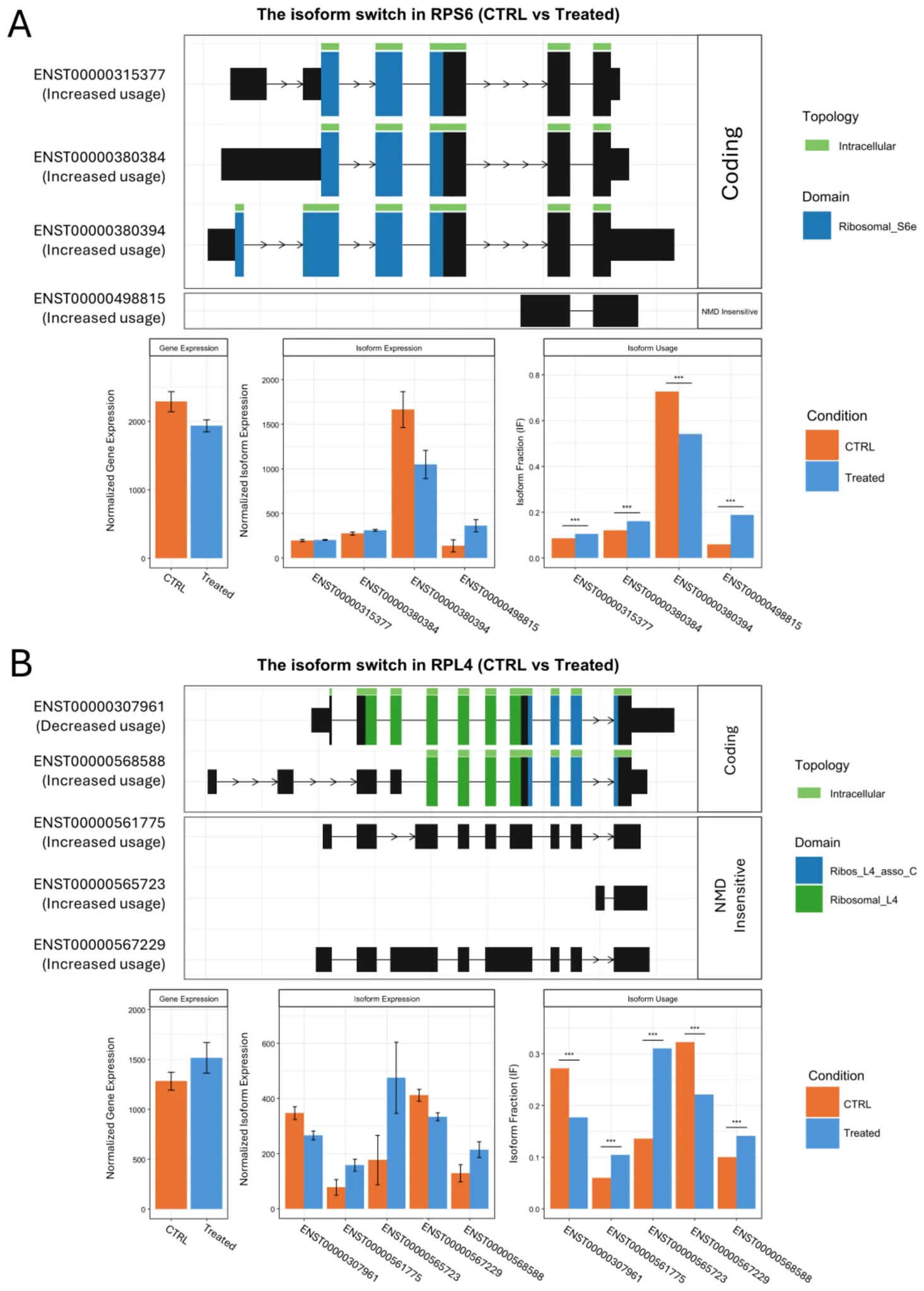
Switch plots of representative translation-related genes affected by isoform switching. (**A**) RPS6; (**B**) RPL4. For each gene, the left panel shows normalized gene expression, the middle panel shows normalized isoform expression, and the right panel shows isoform fraction (IF), defined as the relative contribution of each isoform to total gene expression. Control and radiotherapy-treated cells are shown separately. Transcript structures, predicted coding regions, protein domains, and transmembrane topology are shown when available. (*** = adjusted *p*-value < 0.001).

RPS6 showed reduced usage of the protein-coding transcript ENST00000380394 and increased usage of the NMD-insensitive non-coding transcript ENST00000498815 after radiotherapy (Figure 5A). RPL4 showed increased usage of the NMD-insensitive non-coding transcript ENST00000565723 and relatively higher usage of the protein-coding transcript ENST00000307961 in control cells (Figure 5B).

**Figure 6:**
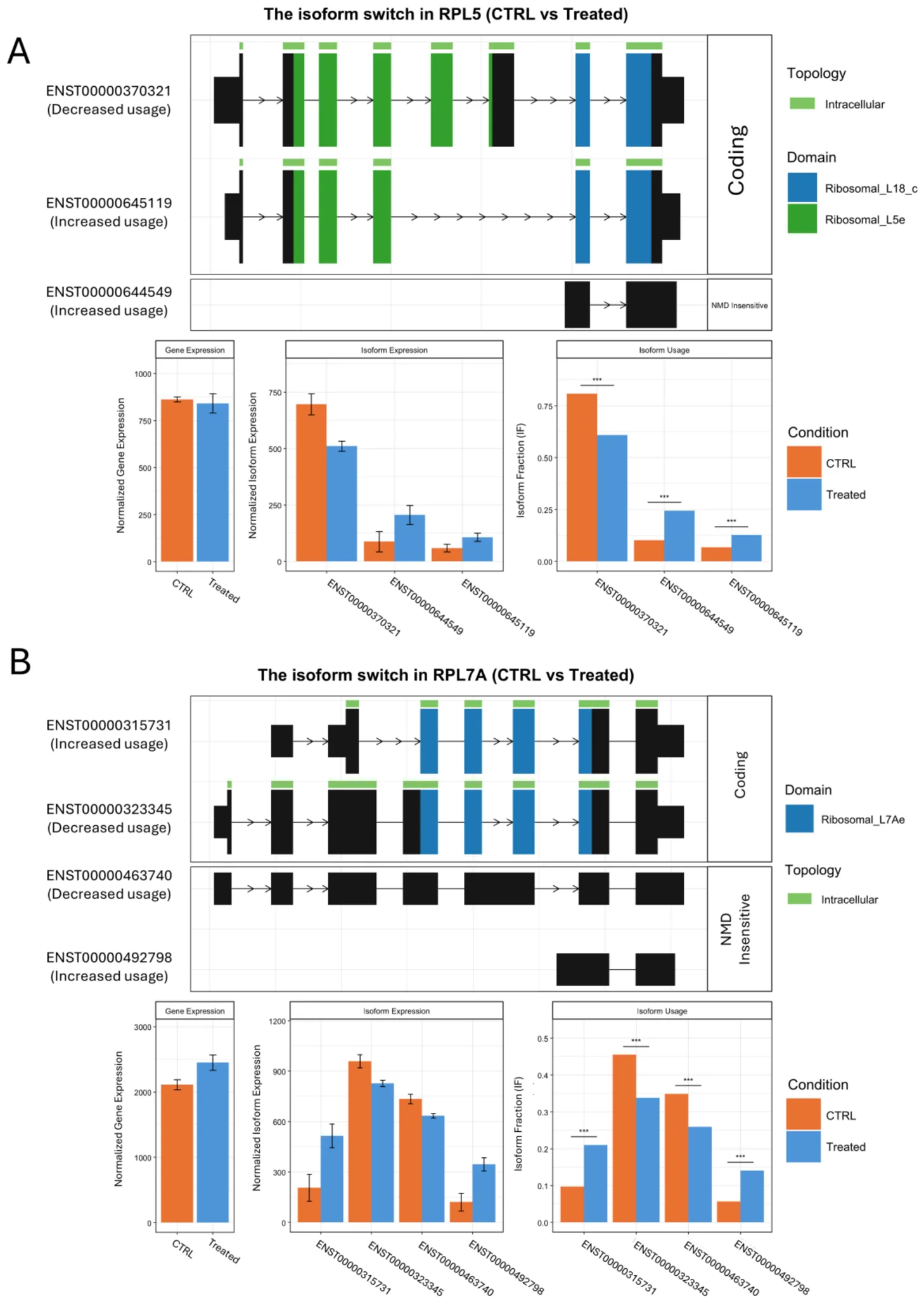
Switch plots of representative translation-related genes affected by isoform switching. (**A**) RPL5; (**B**) RPL7A. For each gene, the left panel shows normalized gene expression, the middle panel shows normalized isoform expression, and the right panel shows isoform fraction (IF), defined as the relative contribution of each isoform to total gene expression. Control and radiotherapy-treated cells are shown separately. Transcript structures, predicted coding regions, protein domains, and transmembrane topology are shown when available. (*** = adjusted *p*-value < 0.001).

RPL5 showed reduced usage of the protein-coding transcript ENST00000370321 and increased usage of the NMD-insensitive non-coding transcript ENST00000644549 after radiotherapy (Figure 6A). RPL7A showed reciprocal changes among four major transcripts, including protein-coding and NMD-insensitive non-coding isoforms (Figure 6B).

**Figure 7:**
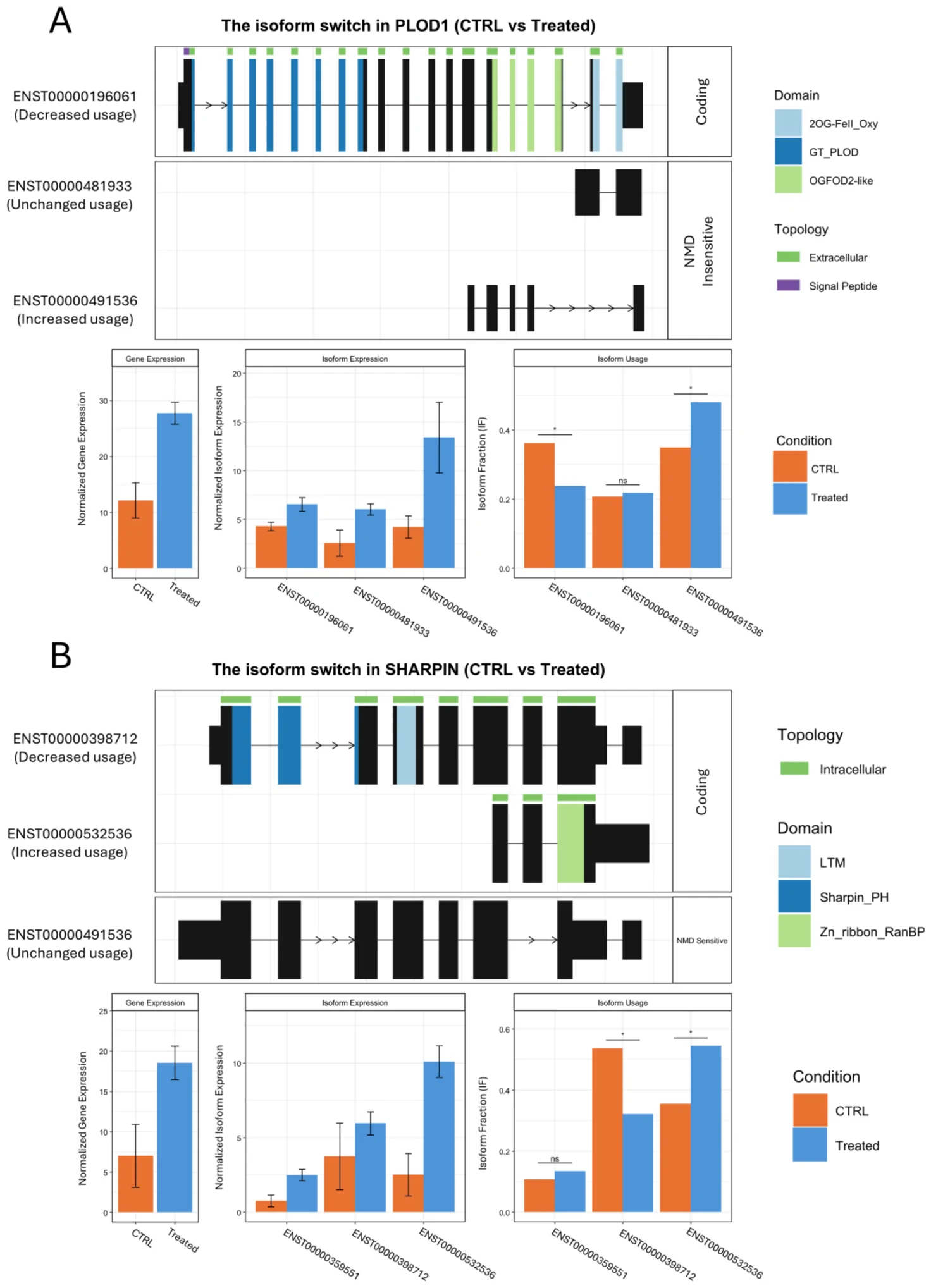
Switch plots of representative non-translation pathway genes affected by isoform switching. (**A**) PLOD1; (**B**) SHARPIN. For each gene, the left panel shows normalized gene expression, the middle panel shows normalized isoform expression, and the right panel shows isoform fraction (IF), defined as the relative contribution of each isoform to total gene expression. Control and radiotherapy-treated cells are shown separately. Transcript structures, predicted coding regions, protein domains, and transmembrane topology are shown when available. (ns = not significant; * = adjusted *p*-value < 0.05).

**Figure 8:**
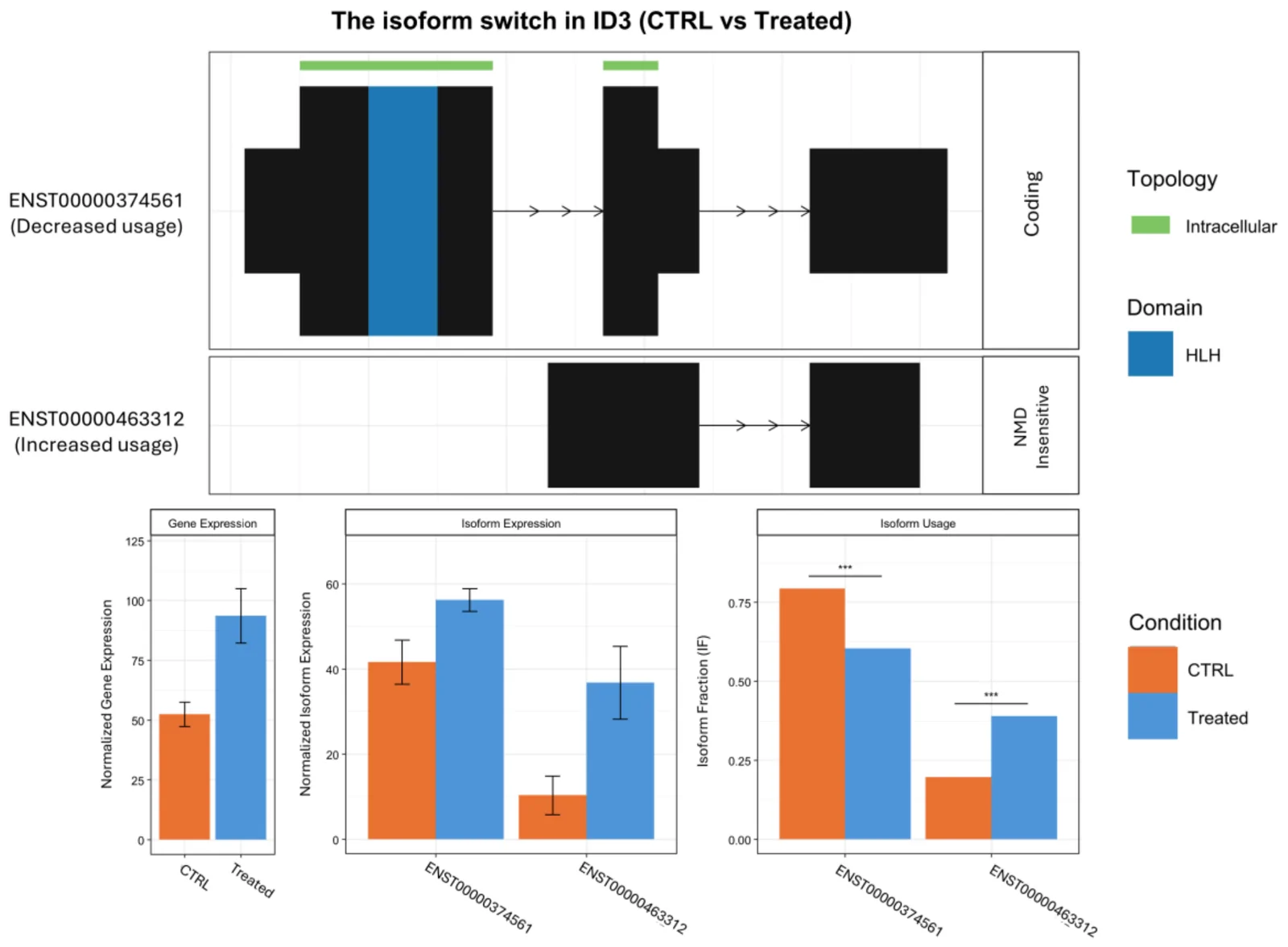
Switch plot of ID3, a representative gene associated with NTRK signaling and affected by isoform switching. The left panel shows normalized gene expression, the middle panel shows normalized isoform expression, and the right panel shows isoform fraction (IF), defined as the relative contribution of each isoform to total gene expression. Control and radiotherapy-treated cells are shown separately. (*** = adjusted *p*-value < 0.001).

Among positively enriched non-translation pathways, PLOD1 showed increased usage of ENST00000491536 after radiotherapy, SHARPIN showed increased usage of ENST00000532536, and ID3 showed increased usage of the NMD-insensitive transcript ENST00000463312 (Figure 7 and Figure 8).

**Figure 9:**
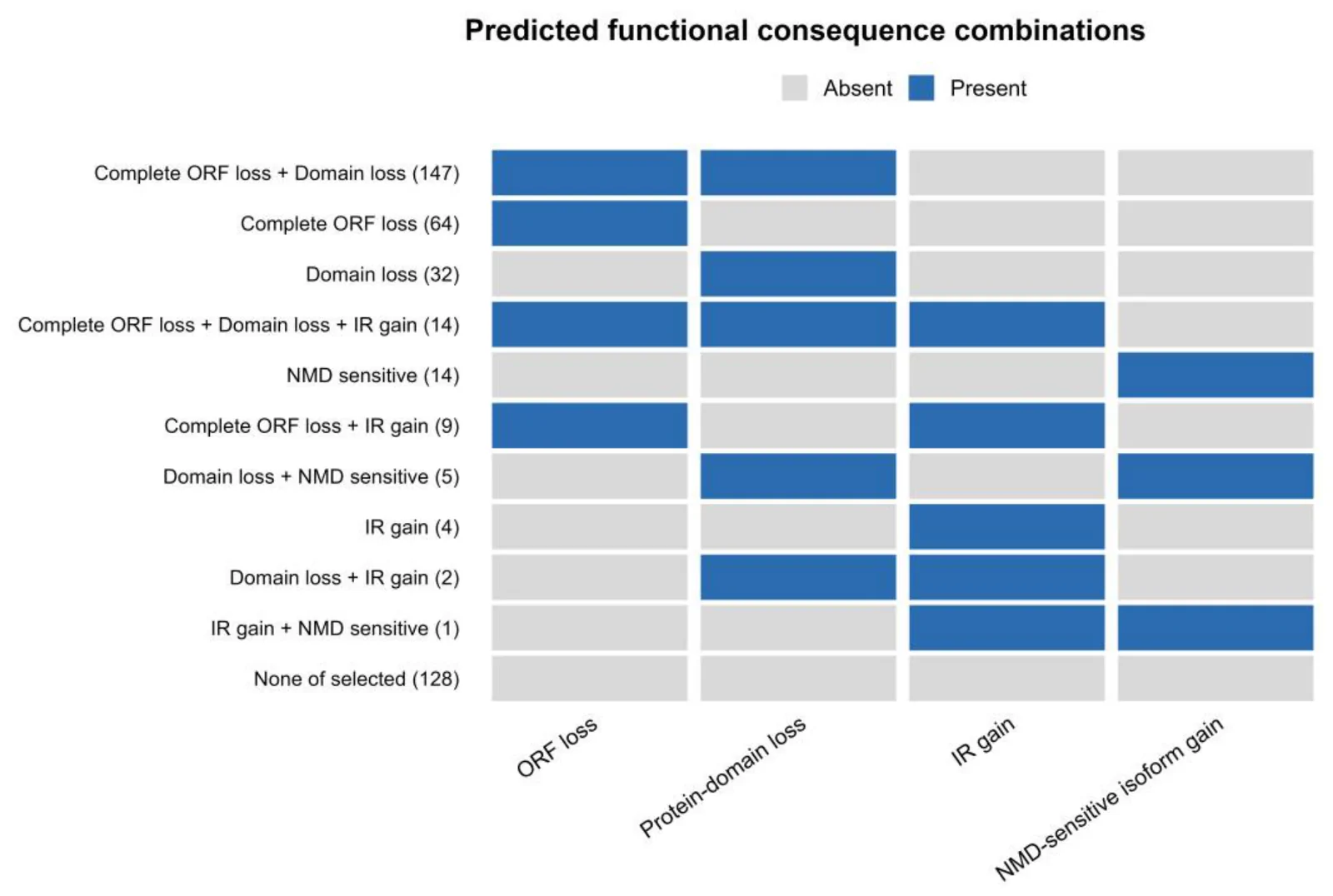
Predicted functional consequence combinations associated with significant isoform-switching events. Rows indicate the most frequent combinations of predicted consequences, including open reading frame loss, protein-domain loss, intron-retention gain, and increased usage of NMD-sensitive isoforms. Blue cells indicate the presence of the corresponding consequence, whereas gray cells indicate its absence. The number of switching events assigned to each combination is reported in parentheses.

Predicted functional consequences were then assessed for significant switches using coding potential (CPAT), open reading frame, protein-domain (PFAM), intron-retention, and NMD-related annotations. The most frequent consequence combination was concurrent ORF loss and protein-domain loss, observed in 147 switching events. ORF loss alone was detected in 64 events, whereas domain loss alone was detected in 32. Fourteen switches showed increased usage of NMD-sensitive isoforms, 14 combined ORF loss, domain loss, and intron-retention gain, and 9 combined ORF loss with intron-retention gain. Additional, less frequent consequence patterns included domain loss with NMD sensitivity, intron-retention gain alone, domain loss with intron-retention gain, and intron-retention gain with NMD sensitivity. All these predicted functional consequences are shown in Figure 9. Fisher’s exact test indicated a strong association between ORF loss and protein-domain loss (odds ratio = 8.26, 95% CI: 5.19–13.38; *p* < 2.2 × 10^−16^), suggesting that these predicted consequences frequently co-occurred. However, these annotations represent predicted transcript-level consequences and should not be interpreted as direct evidence of altered protein abundance or function.

### 2.6. Proteomic Analysis Supports Radiotherapy-Associated Remodeling of the Translation Machinery

To determine whether the transcript-level remodeling observed after radiotherapy was accompanied by changes in protein abundance, we performed an exploratory proteomic analysis in control and irradiated HMC3 cells. Protein abundance was quantified in three biological replicates per condition, and differential abundance was assessed using a limma-based statistical framework with Benjamini–Hochberg correction for multiple testing.

**Figure 10:**
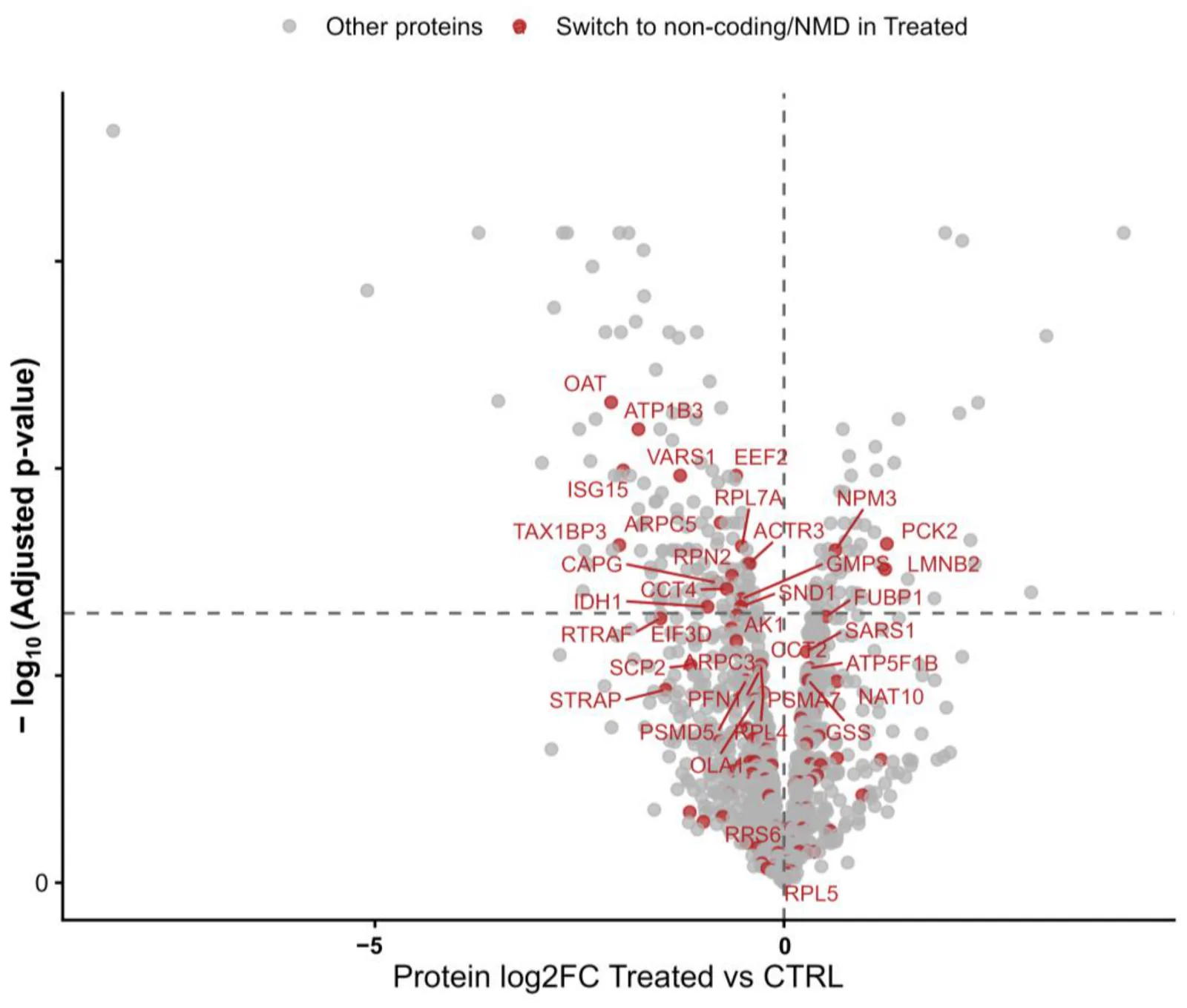
Proteomic changes associated with genes undergoing isoform switching after radiotherapy. Volcano plot showing differential protein abundance in radiotherapy-treated versus control HMC3 cells. The *x*-axis represents protein log2 fold change, and the *y*-axis represents −log10 of the Benjamini–Hochberg-adjusted *p*-value obtained using limma. The horizontal dashed line indicates an adjusted *p*-value of 0.05, while the vertical dashed line indicates a log2 fold change of 0. Red points indicate proteins encoded by genes showing increased usage of at least one non-coding, retained-intron, or NMD-associated transcript after radiotherapy; gray points represent all other quantified proteins.

Among the 1157 quantified proteins, 191 showed significantly altered abundance after radiotherapy at an adjusted *p*-value < 0.05. Of these, 131 displayed negative log2 fold changes, and 60 displayed positive log2 fold changes in irradiated versus control cells. Among the proteins with reduced abundance, 15 were encoded by genes showing increased usage of at least one non-coding, retained-intron, or NMD-associated transcript after radiotherapy. Three proteins with increased abundance were encoded by genes showing the same type of transcript remodeling. These results are summarized in Figure 10.

Full results regarding differential protein abundance are listed in Supplementary Table S6.

**Figure 11:**
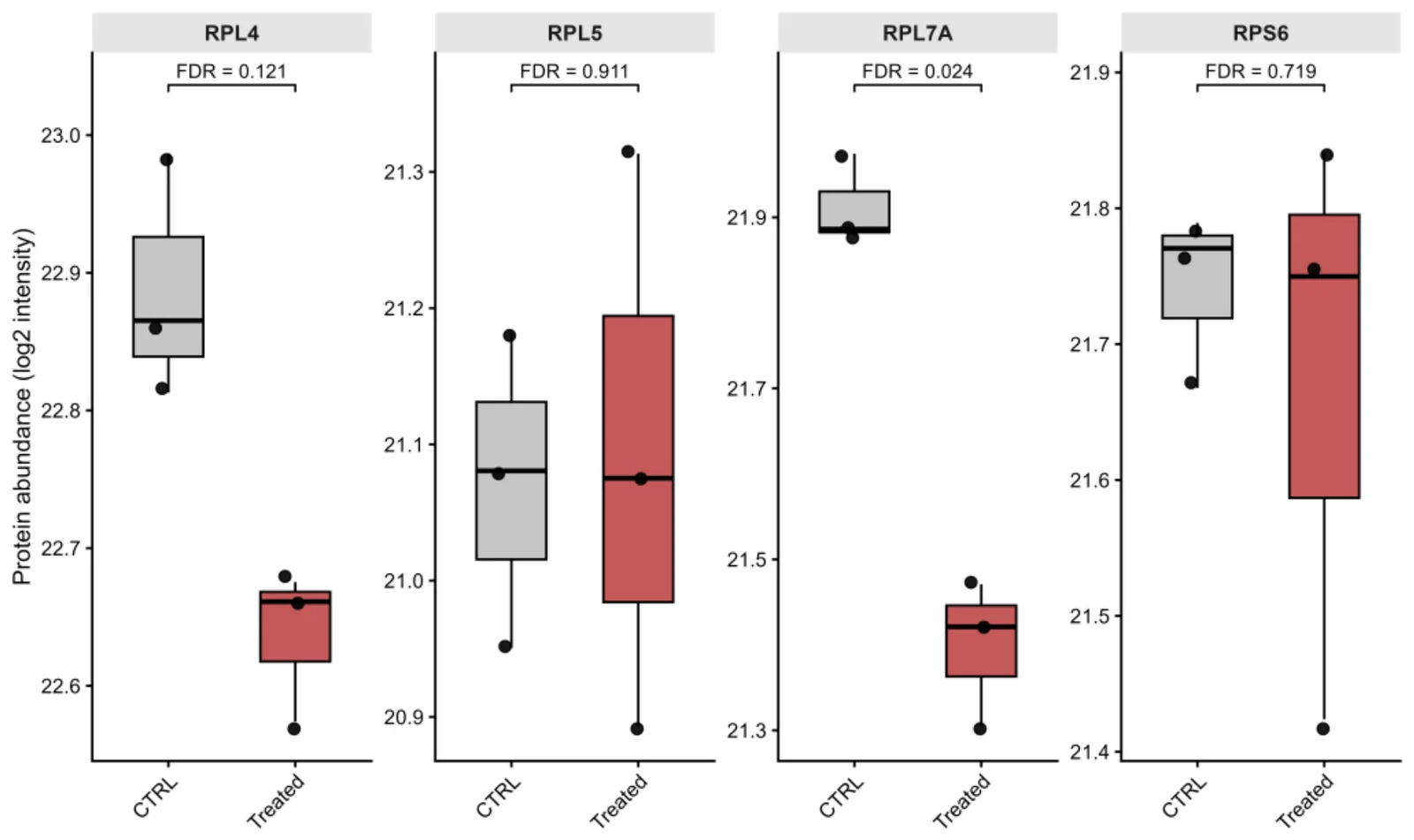
Protein abundance of selected translation-related proteins in control and irradiated HMC3 cells. Boxplots show the log2-transformed normalized protein intensities of RPL4, RPL5, RPL7A, and RPS6 in control and radiotherapy-treated HMC3 cells. Individual dots represent biological replicates (*n* = 3 per condition), central lines indicate medians, and boxes represent the interquartile range. Statistical significance was assessed using the limma moderated *t*-test, with Benjamini–Hochberg correction for multiple testing across the proteomic dataset. RPL7A showed significantly reduced abundance after radiotherapy, whereas RPL4, RPL5, and RPS6 did not remain significant after proteome-wide multiple-testing correction.

We next examined RPS6, RPL4, RPL5, and RPL7A, which were selected because they belonged to negatively enriched translation-related pathways and showed significant changes in isoform usage. RPL7A protein abundance was significantly reduced after radiotherapy following proteome-wide multiple-testing correction (FDR = 0.024). RPL4 also showed reduced abundance, with a nominal *p*-value of 0.039, although this association did not remain significant after correction (FDR = 0.121). No statistically significant differences were observed for RPS6 or RPL5 (Figure 11).

## 3. Discussion

The findings of this study underscore an important shift in our understanding of the microglial response to radiotherapy, moving from a simple model of gene-level activation to a complex, isoform-centric functional modification. By utilizing LR-RNA-seq, we have demonstrated that the microglial transcriptome undergoes significant structural reorganization following radiotherapy, a process often invisible to traditional short-read sequencing technologies [3,23]. From a clinical perspective, the speed and resolution provided by LR-RNA-seq platforms are increasing the importance of this methodology; these technologies can identify complex genomic alterations and variants, offering a potential pathway for molecular monitoring of patients for diagnosis and prognosis in a wide variety of clinical fields [24]. This high-resolution mapping is particularly critical in neuro-oncology, where the interplay between the tumor and the irradiated normal parenchyma dictates long-term neurological outcomes [13].

Although direct longitudinal sampling of microglial tissue is not clinically feasible, previous studies provide proof-of-principle that transcript- and isoform-related signals can be monitored over time in accessible patient-derived samples. Serial analysis of circulating tumor cells in prostate cancer demonstrated that splice-variant status can change dynamically during therapy and can be repeatedly assessed through blood-based sampling [25]. In glioblastoma, longitudinal profiling of serum-derived extracellular-vesicle RNA showed that circulating RNA signatures can distinguish patients from healthy controls and may reflect therapeutic response [26]. In parallel, Oxford Nanopore-based long-read sequencing has been applied to longitudinally track leukemia subclones, fusion transcripts, and isoform landscapes at single-cell resolution [27].

Our results demonstrate that while RT induces significant differential gene expression, one of the most profound functional changes occurs at the isoform level, where genes frequently switch toward non-coding or truncated variants characterized by the loss of functional protein domains.

Importantly, the integration of transcript-level DESeq2 differential abundance with IsoformSwitchAnalyzeR highlighted the complementary nature of these two analytical layers. A substantial fraction of significant isoform-switching events was not classified as differentially abundant at the transcript level by DESeq2. This observation is consistent with the conceptual distinction between absolute transcript abundance and relative isoform usage within a gene. Thus, transcript-level differential abundance and isoform-switching analysis provide non-redundant information: the former captures changes in transcript abundance, whereas the latter detects remodeling of isoform composition that may occur even in the absence of large absolute expression changes.

Previous transcriptomic studies of irradiated microglia identified aging-like changes and persistent innate immune reprogramming, including inflammatory and priming-associated features [10,14]. The present results only partially overlap with these previously reported responses. At the gene-expression level, irradiated HMC3 cells showed enrichment of pathways related to extracellular matrix organization, receptor signaling, neuronal communication, and cytokine-associated processes, together with negative enrichment of translation, ribosome biogenesis, DNA replication, and mitochondrial respiratory pathways. These alterations are compatible with a broader stress-associated and remodeling response, although they do not establish the presence of a complete canonical aging-like or inflammatory state.

These findings are also consistent with previous evidence linking defective G2/M checkpoint regulation to genomic instability under genotoxic stress conditions [28].

Such stress-associated remodeling may also intersect with cytoprotective pathways involved in oxidative and inflammatory responses, including the heme oxygenase-1/carbon monoxide axis, which has been implicated in vascular protection and inflammatory injury responses [29].

Long-read RNA sequencing provides a complementary layer of information by resolving transcript diversity and relative isoform usage within individual genes [2]. In the present study, radiotherapy-associated isoform remodeling was observed even in genes whose total expression remained relatively stable or changed in a direction that did not fully reflect the behavior of individual transcripts. Similar dissociation between gene-level abundance and transcript-specific regulation has been observed in long-read studies of microglial and neurological transcriptomes and in analyses of the response to ionizing radiation [2,5,18]. Therefore, the aging-like and inflammatory patterns previously identified through gene-level transcriptomic analyses and the isoform-switching events described here should be viewed as complementary, rather than directly equivalent, components of the microglial response to RT.

At the gene-set level, positively enriched PI3K/AKT-related pathways were observed after radiotherapy. However, this result reflects the distribution of pathway-associated genes within the ranked expression list and should not be interpreted as direct evidence of increased AKT protein activity or phosphorylation. In the context of the brain tumor microenvironment, PI3K/AKT signaling has been implicated in the regulation of microglial phenotypes [30]. Specifically, radiation-induced AKT phosphorylation has been shown to increase the expression of anti-inflammatory/immunosuppressive markers and decrease pro-inflammatory markers [31]. This shift is of critical relevance within the tumor since it may promote a tumor-favorable microenvironment [7].

Our finding that this pathway remains positively enriched post-RT aligns with previous reports of sustained glial activation intended to repair radiation-induced damage, which may inadvertently support tumor growth [32,33].

A major finding of this study is the downregulation of translation mechanisms at the gene level, a hallmark of the “integrated stress response” where cells attenuate global protein synthesis to prioritize survival and repair [20,34]. The “ribostress” and translational reprogramming observed through isoform switching in ribosomal proteins suggest that the cognitive decline often reported by patients may contribute to a qualitative loss of functional protein domains rather than a simple reduction in cell number [11,16,19].

Four representative translation-related genes were selected as the most overlapping with the gene set enrichment analysis: RPS6, RPL4, RPL5, and RPL7A. These genes displayed reciprocal changes in the usage of protein-coding and non-coding or NMD-related transcripts. Such alterations are consistent with a transcriptomic pattern suggestive of ribosomal stress and translational reprogramming. Nevertheless, predicted changes in coding potential, open reading frames, or protein domains do not by themselves demonstrate impaired ribosome function or reduced protein synthesis. Such isoform remodeling may represent one mechanism through which cells modulate coding output during stress, although this interpretation requires isoform-specific experimental validation [35].

The proteomic analysis provided an important orthogonal layer for interpreting these transcriptomic findings. Among 1157 quantified proteins, 191 showed significantly altered abundance after radiotherapy, with 131 displaying negative and 60 positive log2 fold changes. Fifteen proteins with reduced abundance were encoded by genes showing increased usage of at least one non-coding, retained-intron, or NMD-associated transcript, whereas three proteins with increased abundance were associated with the same type of transcript remodeling. This partial correspondence indicates that isoform-level changes may accompany altered protein abundance in a subset of genes but also demonstrates that transcript remodeling does not uniformly translate into detectable changes in total protein levels.

Among the selected translation-related candidates, RPL7A showed a significant reduction in protein abundance after proteome-wide multiple-testing correction. This result provides the strongest protein-level support for the transcriptomic alterations affecting ribosomal genes. RPL4 also showed reduced protein abundance and a nominally significant difference, although it did not remain significant after correction for multiple testing. In contrast, RPS6 and RPL5 did not show significant protein-level changes. These results argue against a simple one-to-one relationship between isoform switching and total protein abundance and suggest that the consequences of transcript remodeling may depend on transcript stability, translation efficiency, protein turnover, and the ability of the proteomic workflow to detect isoform-specific products.

The predicted functional-consequence analysis showed a strong association between open-reading-frame loss and protein-domain loss. This indicates that these computationally predicted consequences frequently co-occurred among significant switching events. However, this association should not be interpreted as evidence that the corresponding non-functional proteins were produced. Rather, it identifies a subset of transcript switches with a higher predicted potential to alter coding output and provides a basis for prioritizing future experimental validation.

Our findings are conceptually consistent with recent transcript-level profiling of ex vivo human microglia in Alzheimer’s disease, which showed that disease-associated transcript heterogeneity can reveal regulatory changes missed by gene-level analyses, including truncated NMD-predicted isoforms. Although that study differs from ours in disease context, cellular model and sequencing strategy, it supports the broader concept that microglial dysfunction may involve transcript- or isoform-specific remodeling beyond changes in total gene abundance. In this sense, our LR-RNA-seq data extend this concept to radiation-induced stress, showing that irradiated microglia undergo isoform switching toward non-coding or potentially non-functional transcript variants [36].

In addition to translation-associated genes, isoform switching was observed in genes linked to extracellular matrix organization, neuronal signaling, and receptor pathways, including PLOD1, SHARPIN, and ID3. PLOD1 has been associated with extracellular matrix remodeling and glioma progression [37,38], while SHARPIN and ID3 participate in signaling processes relevant to cellular stress and tissue remodeling. In the present study, these genes should be regarded as representative examples of pathway-associated isoform remodeling rather than as validated mediators of tumor progression or radiation-induced neurotoxicity. The shift toward specific isoforms of these genes in microglial cells suggests that even normal-appearing brain parenchyma can be reprogrammed by RT to express biomarkers typically associated with malignant progression [9,39].

Translating these molecular insights into clinical practice requires a focus on therapeutic modulation of the microglial phenotype. The shift toward tumor-supportive or dysfunctional isoforms identified in our study provides a mechanistic target for interventions aimed at preserving cognitive function. For instance, the use of CSF1R inhibitors to deplete and subsequently repopulate the brain with “fresh” microglia has shown promise in mitigating radiation-induced injury by resetting the inflammatory environment [40]. Furthermore, targeting specific signaling axes like the MIF-CD74 pathway could theoretically prevent the transition toward tumor-favorable states, instead promoting a pro-inflammatory phenotype that synergizes with radiotherapy to eliminate residual tumor cells [7]. By identifying specific isoform-level biomarkers in the blood or cerebrospinal fluid, clinicians might be able to detect the earliest signs of radiation-induced brain injury before irreversible structural damage or cognitive impairment manifests [41]. Ultimately, this research may help bridge basic transcriptomics and clinical application, suggesting that the molecular state of the normal brain could be monitored and potentially protected through isoform-targeted precision medicine [12,42].

Despite the advantages of LR-RNA-seq, several limitations should be considered. First, the study was conducted exclusively in the immortalized HMC3 cell line. Although HMC3 cells represent a widely used human microglial model, they may not fully reproduce the transcriptional, phenotypic, and functional properties of primary human microglia or their interactions with other central nervous system cell types [43]. Therefore, the isoform-switching patterns identified here should be regarded as specific to the HMC3 model under the experimental conditions tested and should not be generalized to primary or in vivo microglia without independent validation.

Second, although the 24-h transcriptome has been shown to be strikingly similar to the chronic 1-month signature in irradiated microglia [14], the study was conducted using a single radiation dose, a single post-irradiation time point, and three biological replicates per condition, limiting the assessment of dose-dependent and temporal changes.

The present study was primarily designed to characterize radiotherapy-associated transcriptomic remodeling, including changes in transcript abundance, isoform usage, and predicted transcript-level consequences. Although the proteomic analysis provided partial orthogonal support for selected translation-associated alterations, it quantified total protein abundance and did not resolve isoform-specific protein products. Moreover, no direct phenotypic assays were performed to determine whether the observed molecular changes were associated with cell death, growth inhibition, altered proliferation, inflammatory activation, or other microglial functional outcomes. Therefore, the identified isoform-switching events should not be interpreted as direct evidence of microglial dysfunction.

Future studies should combine isoform-specific validation with functional assays and independent cellular models, including primary human microglia or induced pluripotent stem cell-derived microglia. Longitudinal and dose–response experiments will also be required to establish whether the observed transcriptomic remodeling is transient, persistent, or linked to specific phenotypic consequences after irradiation.

## 4. Materials and Methods

### 4.1. Cell Culture and Treatment

The human microglia cell line (HMC3) was purchased from ATCC Company (Milan, Italy). HMC3 cells were cultured according to the recommendations by ATCC, where EMEM (ATCC^®^ 30–2003TM) was used as the base medium and completed by adding 56 mL FBS (ATCC^®^ 30–2020TM) to 500 mL of base EMEM [44]. Experiments were performed in triplicate biological replicates (*n* = 3 independent cell cultures per condition). Radiotherapy was delivered using a 6 MV X-ray beam from an Elekta Synergy linear accelerator (Elekta AB, Stockholm, Sweden) (Radiotherapy Department, Cannizzaro Hospital, Catania, Italy) at a dose rate of 3 Gy/min. HMC3 cells were exposed to 0 Gy (control) or 15 Gy (treated). After RT, cells were incubated at 37 °C in a humidified atmosphere of 5% CO_2_ for 24 h [13].

### 4.2. RNA Extraction and Library Preparation

Total RNA was extracted using the RNeasy Mini Kit (Qiagen, Germantown, MD, USA, 74104). RNA integrity was assessed using the Agilent 2100 Bioanalyzer with the Eukaryote Total RNA Nano 6000 Assay (Agilent, Santa Clara, CA, USA, 5067-1511), and quantification was performed using the Qubit RNA Broad Range Assay Kit (Invitrogen™, ThermoFisher, Carlsbad, CA, USA, Q32851), with 500 ng of total RNA used as input for library preparation.

Full-length cDNA libraries were prepared using the Oxford Nanopore Technologies (ONT) cDNA-PCR Barcoding Kit V14 (SQK-PCB114.24, Oxford Nanopore Technologies, Oxford, UK). The protocol included a reverse transcription step with strand-switching primers, enabling capture of full-length polyadenylated transcripts and incorporation of unique molecular identifiers (UMIs) to enhance isoform-level resolution.

Reverse transcription was followed by PCR amplification using rapid attachment barcode primers, allowing multiplexing of up to 24 samples without the need for ligation-based adapter attachment. After 40 min of amplification, a bead-based clean-up was performed to remove short cDNA fragments. The barcoded PCR products were then pooled, and sequencing adapters were ligated using the Rapid Adapter Mix in a 5 min reaction. The final libraries were loaded onto PromethION flow cells (Oxford Nanopore Technologies, Oxford, UK, FLO-PROMIN114) and sequenced using MinKNOW software (Version 24.11.11, Oxford Nanopore Technologies, Oxford, UK). Sequencing was conducted on R10.4.1 flow cells using Kit 14 chemistry, optimized for high accuracy (Q20+) and efficient isoform detection.

### 4.3. Bioinformatic Analyses

Data generated during the sequencing were basecalled with MinKNOW (https://nanoporetech.com/document/experiment-companion-minknow, accessed in 23 March 2025). The obtained reads were processed using the Epi2me Labs (https://github.com/epi2me-labs, accessed in 23 March 2025), “wf-transcriptome pipeline”. The alignment to the reference genome was performed using Minimap2 [45], followed by transcript assembly (StringTie2) [46], annotation (GffCompare) [47], and quantification (Salmon) [48]. Differential gene expression and transcript usage were analyzed with DESeq2 [49], DRIMSeq [50], and DEXSeq [51], with stageR [52] applied for multi-stage false discovery rate control.

A minimum of 3 million reads per sample was obtained, with an average read length of 910 bp and a median Q-score > 12.

Differential expression analysis was performed at both gene and transcript level using DESeq2. For the gene-level analysis, counts assigned to the same gene were summarized to estimate total gene abundance. For the transcript-level analysis, each annotated transcript isoform was retained as an independent molecular feature and tested separately between irradiated and control HMC3 microglial cells. For differential transcript usage analysis, genes were retained when their summed transcript counts reached at least 10 raw counts in at least three samples. Individual transcripts were retained when they reached at least three raw counts in at least one sample. Genes represented by fewer than two retained transcripts were subsequently excluded. The resulting matrix contained 74,843 transcripts assigned to 11,118 genes. Differential expression or differential transcript abundance was assessed using the Wald test, and *p*-values were adjusted for multiple testing using the Benjamini–Hochberg method. Features with an adjusted *p*-value < 0.05 and |log2 fold change| > 1 were considered significantly differentially expressed or differentially abundant.

Gene set enrichment analysis was performed using Reactome gene sets and a pre-ranked gene list derived from the DESeq2 gene-level analysis. Genes were ranked according to the DESeq2 Wald statistic. Therefore, GSEA considered the distribution of all ranked genes within each pathway, rather than only genes passing the adjusted *p*-value and log2 fold-change thresholds used to define differentially expressed genes in the volcano plot.

Differential isoform expression and isoform switch analysis were performed using the IsoformSwitchAnalyzeR package (Version 2.12.0) [53]. Isoform fraction (IF) was calculated as the abundance of each transcript divided by the total transcript abundance of the corresponding gene. Delta Isoform Fraction (dIF) was defined as IF_Treated − IF_CTRL. An isoform switch was defined as a significant change in isoform fraction with an isoform-switch q-value < 0.05 and |dIF| ≥ 0.1 between conditions, as implemented in IsoformSwitchAnalyzeR. To functionally characterize genes affected by isoform switching, Reactome pathway enrichment analysis was performed. GeneRatio was defined as the number of input genes annotated to a given Reactome pathway divided by the total number of input genes used for enrichment analysis.

### 4.4. Proteomic Analysis by LC-MS

Sample preparation for MS-based proteomic analysis was carried out as previously reported [54] with minor modifications. The cell pellets were sonicated at 4 °C for 2 min in a lysis buffer (RIPA), containing protease inhibitors (10 μL/mg) and incubated. After centrifugation at 13,000× *g* for 10 min at 4 °C, the protein content was precipitated from the supernatants by combining them with prechilled acetone (−20 °C). The mixtures were centrifuged at 10,000× *g*, and the pellet was resuspended in 8 M urea. The protein concentration was quantified via the BCA protein assay kit (Thermo Fisher Scientific, Waltham, MA, USA). The protein sulfhydryl groups were reduced and alkylated using DTT (1.6 mM) and iodoacetamide (7 mM), respectively. Samples were diluted 1:10 with 50 mM NH_4_HCO_3_ and treated with trypsin at 37 °C for 15 h (protein-to-trypsin ratio being 50:1 *w*/*w*). The hydrolytic peptides were separated and detected via a UHPLC system (Thermo Fisher Scientific Dionex UltiMate 3000 RSLCnano) coupled with an Orbitrap Fusion Tribrid mass spectrometer (Q-OT-qIT, Thermo Fisher Scientific, Waltham, MA, USA). An aqueous solution containing 5% formic acid (FA) was used to dilute the samples. Peptides were eluted on a PepMap^®^ RSLC C18 column (EASYSpray, 75 μm × 50 cm, 2 μm, 100 Å, Thermo Fisher Scientific, Waltham, MA, USA) and separated by elution at a flow rate of 0.250 μL/min at 40 °C, with a linear gradient of solvent B (CH_3_CN + 0.1% FA) in solvent A (H_2_O + 0.1% FA). The eluted peptides were ionized via a nanospray (Easy-spray ion source, Thermo Scientific, Waltham, MA, USA) using a capillary temperature and voltage set to 275 °C and 2.0 kV, respectively. Peptide precursor scans in the *m*/*z* range of 400–1600 were performed with a resolution of 120,000 (200 *m*/*z*) with an AGC target for Orbitrap detection of 4.0 × 10^5^ and a maximum injection time of 50 ms. MS/MS spectra were acquired using a normalized collision energy (HCD) of 35. The dynamic exclusion duration was set to 60 s with a tolerance of 10 ppm around the selected precursor and its isotopes. The AGC target values and maximum injection times (ms) for the MS/MS spectra were 1.0 × 10^4^ and 100, respectively. The mass spectrometer was calibrated with Pierce^®^ LTQ Velos ESI positive ion calibration solution (Thermo Fisher Scientific, Waltham, MA, USA). MS data acquisition was performed via Xcalibur software v. 3.0.63 (Thermo Fisher Scientific, Waltham, MA, USA). The mass spectra were analyzed via MaxQuant software (version 2.8.0.0). The initial maximum allowed mass deviation was set to 6 ppm for monoisotopic precursor ions and 0.5 Da for MS/MS peaks. The enzyme specificity was set to trypsin, defined as the C-terminus to arginine and lysine, excluding proline, with a maximum of 2 missed cleavages allowed. Carbamidomethyl cysteine was set as a fixed modification, whereas N-terminal acetylation and methionine oxidation were set as variable modifications. The spectra were analyzed via the Andromeda search engine against the *Homo sapiens* UniProt sequence database (11 June 2026). Quantification in MaxQuant was performed via the integrated Label-Free Quantification (LFQ) algorithm, which is based on extracted ion chromatograms (XIC) and employs fast LFQ. To facilitate the intersection between proteomics and transcriptomics, proteins were labeled as the genes encoding them. Differential protein abundance analysis was carried out using a Limma-integrated approach [55], and a statistical significance cutoff was set to an adjusted *p*-value level of <0.05 to consider a protein as differentially abundant between treated and control HMC3 cells.

The mass spectrometry proteomics data have been deposited to the ProteomeXchange Consortium via the PRIDE [56] partner repository with the dataset identifier PXD081768.

## 5. Conclusions

This study identifies coordinated gene-, transcript-, and isoform-level changes in irradiated HMC3 microglial cells. Long-read RNA sequencing revealed significant remodeling of relative isoform usage, particularly in genes involved in translation, ribosome biology, mitochondrial respiration, and cell-cycle regulation. The integration of transcriptomic and proteomic data provided partial protein-level support for selected translation-associated alterations, including a significant reduction in RPL7A abundance.

These findings demonstrate that gene-level expression alone may not fully capture the molecular response of HMC3 cells to radiotherapy. However, the functional consequences of the identified isoform switches remain to be established in independent microglial models and through isoform-specific, biochemical, and phenotypic validation.

## Figures and Tables

**Table 1:** Representative overlap between gene-level Reactome GSEA pathways and genes undergoing significant isoform switching. The table reports selected pathways with positive or negative GSEA normalized enrichment scores (NES) whose core-enrichment genes overlap with genes showing significant isoform switching. Negative NES values indicate enrichment toward the downregulated side of the gene-level ranked list in treated versus control cells, whereas positive NES values indicate enrichment toward the upregulated side. Gained and reduced isoform classes refer to transcript biotype-derived classes of isoforms whose usage increased or decreased after radiotherapy, respectively. The complete gene- and isoform-level overlap is provided in Supplementary Table S5.

Reactome_Pathway	NES	GSEA_Direction	GSEA_FDR	n_Switching_Genes	Switching_Genes	Gained_Isoform_Classes	Reduced_Isoform_Classes	Min_Switch_q_Value
Translation	−3.247	Enriched in control/downregulated side	2.08 × 10^−9^	17	EIF2B2; EIF2S1; EIF3M; EIF4EBP1; MRPL1; MRPL13; MRPL15; MRPL2; MRPL38; RPL4; +7 more	Protein-coding: 12; Retained intron: 6	Protein-coding: 16; Retained intron: 2	7.05 × 10^−8^
Aerobic respiration and respiratory electron transport	−2.786	Enriched in control/downregulated side	2.08 × 10^−9^	15	ATP5F1C; ATP5PB; COX16; DMAC1; GLO1; MPC2; NDUFA9; NDUFAB1; NDUFAF4; NDUFV2; +5 more	Protein-coding: 15; Retained intron: 2; NMD-associated: 1	Protein-coding: 15	7.47 × 10^−7^
Metabolism of amino acids and derivatives	−2.985	Enriched in control/downregulated side	2.08 × 10^−9^	13	APIP; NQO1; PSMA1; PSMA5; PSMA7; PSMB2; PSMB7; RPL4; RPL5; RPL7A; +3 more	Protein-coding: 13; Retained intron: 1	Protein-coding: 12; Retained intron: 2	2.37 × 10^−11^
rRNA processing	−3.271	Enriched in control/downregulated side	2.08 × 10^−9^	11	EBNA1BP2; EMG1; RIOK2; RPL4; RPL5; RPL7A; RPLP1; RPS6; UTP6; WDR12; +1 more	Protein-coding: 9; Retained intron: 3	Protein-coding: 9; Retained intron: 2	1.39 × 10^−8^
DNA replication	−2.382	Enriched in control/downregulated side	2.08 × 10^−9^	11	FEN1; H2BC11; MCM5; PSMA1; PSMA5; PSMA7; PSMB2; PSMB7; PSMD11; RFC4; +1 more	Protein-coding: 11	Protein-coding: 11	2.37 × 10^−11^
Respiratory electron transport	−2.76	Enriched in control/downregulated side	2.08 × 10^−9^	10	COX16; DMAC1; NDUFA9; NDUFAB1; NDUFAF4; NDUFV2; SFXN4; TIMM21; TIMMDC1; UQCRC2	Protein-coding: 9; Retained intron: 2; NMD-associated: 1	Protein-coding: 10	1.10 × 10^−6^
Axon guidance	−2.382	Enriched in control/downregulated side	2.08 × 10^−9^	10	ARPC1A; PFN1; PSMA7; PSMB2; PSMB7; RPL4; RPL5; RPL7A; RPLP1; RPS6	Protein-coding: 9; Retained intron: 2	Protein-coding: 9; Retained intron: 2	2.37 × 10^−11^
Nervous system development	−2.335	Enriched in control/downregulated side	2.08 × 10^−9^	10	ARPC1A; PFN1; PSMA7; PSMB2; PSMB7; RPL4; RPL5; RPL7A; RPLP1; RPS6	Protein-coding: 9; Retained intron: 2	Protein-coding: 9; Retained intron: 2	2.37 × 10^−11^
Extracellular matrix organization	2.294	Enriched in treated/upregulated side	2.08 × 10^−9^	1	PLOD1	Protein-coding: 1	Protein-coding: 1	1.83 × 10^−2^
Neuronal system	1.968	Enriched in treated/upregulated side	2.63 × 10^−6^	1	SHARPIN	Protein-coding: 1	Protein-coding: 1	1.03 × 10^−3^
Signaling by NTRKs	2.108	Enriched in treated/upregulated side	6.91 × 10^−6^	1	ID3	Protein-coding: 1	Protein-coding: 1	4.80 × 10^−4^
RHO GTPase cycle	1.572	Enriched in treated/upregulated side	5.18 × 10^−5^	1	STOM	Protein-coding: 1	Protein-coding: 1	1.94 × 10^−2^
HS-GAG degradation	2.014	Enriched in treated/upregulated side	2.08 × 10^−3^	1	GLB1	Protein-coding: 2		4.37 × 10^−3^
Cytokine signaling in immune system	1.276	Enriched in treated/upregulated side	6.60 × 10^−3^	1	BST2	NMD-associated: 1	Protein-coding: 1	8.37 × 10^−3^
Heparan sulfate/heparin (HS-GAG) metabolism	1.898	Enriched in treated/upregulated side	7.02 × 10^−3^	1	GLB1	Protein-coding: 2		4.37 × 10^−3^
Death receptor signaling	1.568	Enriched in treated/upregulated side	8.77 × 10^−3^	1	SHARPIN	Protein-coding: 1	Protein-coding: 1	1.03 × 10^−3^

## Data Availability

The raw LR-RNA-seq data and processed expression matrices generated in this study have been deposited in the Gene Expression Omnibus (GEO) database under accession number GSE336257 and are publicly available. Proteomics data have been deposited in ProteomeXchange with identifier PXD081768 and are publicly available. Additional processed results are provided in the Supplementary Information.

## Supplementary Materials

The following supporting information can be downloaded at: https://www.mdpi.com/article/10.3390/ncrna12040031/s1, Supplementary Figure S1: Integration of isoform-switching analysis with transcript-level DESeq2 differential abundance; Supplementary Table S1: Gene-level DESeq2 analysis results; Supplementary Table S2: Transcript-level DESeq2 analysis results; Supplementary Table S3: Full list of significant switching genes and transcripts; Supplementary Table S4: Full list of pathways enriched in switching genes; Supplementary Table S5: Complete list of overlapping genes with GSEA enrichment; Supplementary Table S6: Complete Limma results on differential protein abundance.

## Abbreviations

The following abbreviations are used in this manuscript:

RT	Radiotherapy
IF	Isoform Fraction
PCR	Polymerase Chain Reaction
NMD	Nonsense-Mediated Decay
FBS	Fetal Bovine Serum
GSEA	Gene Set Enrichment Analysis
ORF	Open Reading Frame
