# Supplementary material for: Long-Read RNA Sequencing Reveals an Isoform Switching Pattern in Healthy Microglial Cell Lines Exposed to Radiotherapy
Source: Noncoding RNA. 2026 Aug 6;12(4):31. doi: 10.3390/ncrna12040031 (PMC13516098; doi:10.3390/ncrna12040031)
Supplement: Supplementary file 1 [file ncrna-12-00031-s001.zip › Supplementary Figure S1.pdf]

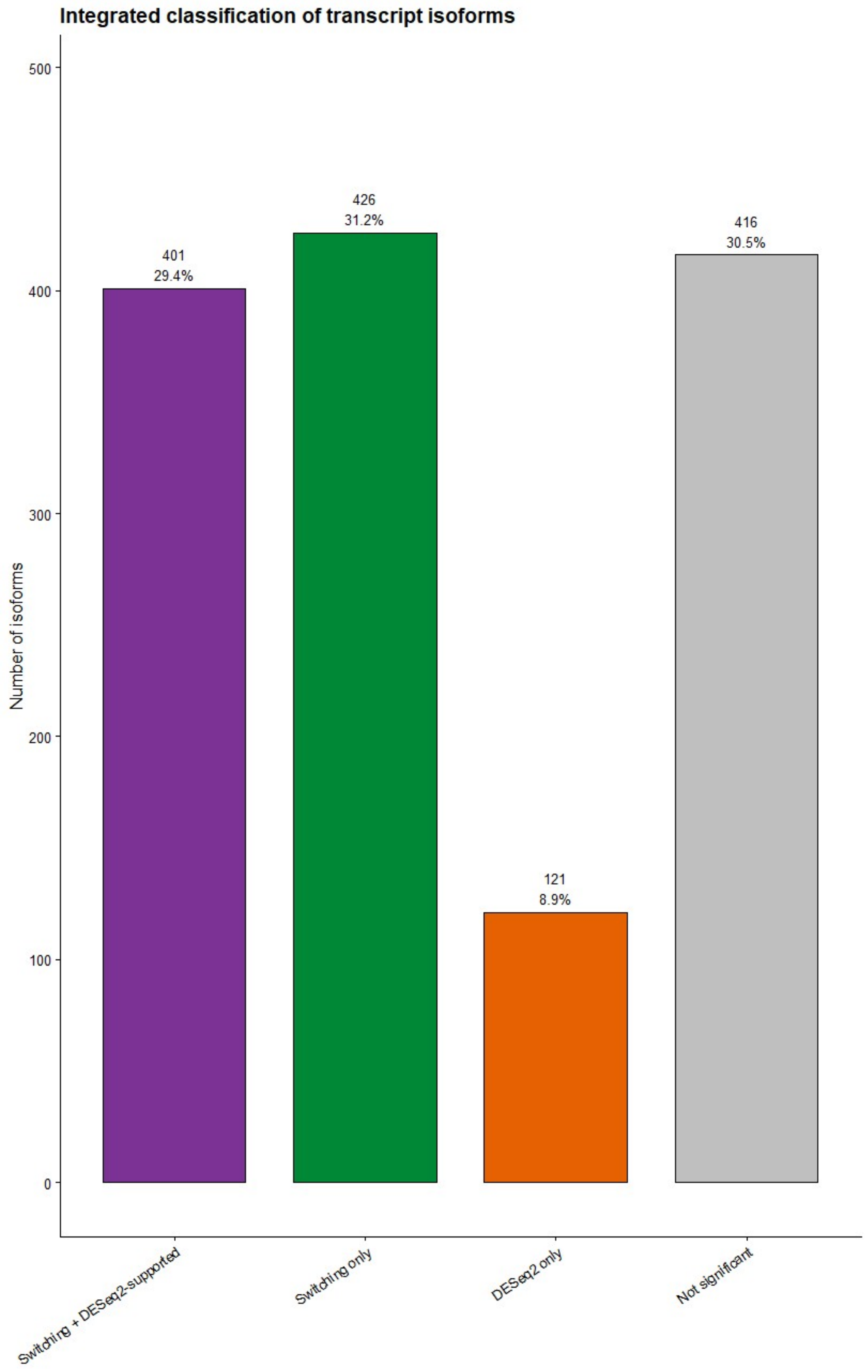

Supplementary Figure S1. Integration of isoform-switching analysis with transcript-level DESeq2 differential abundance. Classification of isoforms according to significant isoform switching and transcript-level DESeq2 differential abundance. "Switching + DESeq2-supported" indicates isoforms showing both significant isoform switching and significant transcript-level differential abundance. "Switching only" indicates isoforms showing significant changes in relative isoform usage without significant transcript-level differential abundance. "DESeq2 only" indicates isoforms significantly differentially abundant at transcript level but not significantly switching.
